# Gonadotropins at Advanced Age - Perhaps They Are Not So Bad? Correlations Between Gonadotropins and Sarcopenia Indicators in Older Adults

**DOI:** 10.3389/fendo.2021.797243

**Published:** 2021-12-24

**Authors:** Agnieszka Guligowska, Zuzanna Chrzastek, Marek Pawlikowski, Malgorzata Pigłowska, Hanna Pisarek, Katarzyna Winczyk, Tomasz Kostka

**Affiliations:** ^1^ Department of Geriatrics, Healthy Ageing Research Centre, Medical University of Lodz, Lodz, Poland; ^2^ Department of Immunoendocrinology, Medical University of Lodz, Lodz, Poland; ^3^ Department of Neuroendocrinology, Chair of Laboratory and Molecular Diagnostics, Medical University of Lodz, Lodz, Poland

**Keywords:** sarcopenia, muscle mass, handgrip strength, TUG, SPPB, LH, FSH, testosterone

## Abstract

Many hormones fluctuate during the aging process. It has been suggested that gonadotropins, which increase with age, contribute to the occurrence of many diseases and syndromes in older life, such as cardiovascular diseases, obesity, frailty syndrome and osteoporosis. This study aims to assess the relationship between circulating gonadotropins and other hormones potentially contributing to age-related functional decline and sarcopenia indicators in 39 male and 61 female community-dwelling seniors, mean age 80 years. According to the definition developed by the second European Working Group on Sarcopenia in Older People (EWGSOP2), the following indicators of the sarcopenia were assessed: bioimpedance-measured body composition, gait speed, handgrip strength, timed up and go test (TUG), chair stand test, Short Physical Performance Battery (SPPB). Blood levels of follicle-stimulating hormone (FSH), luteinizing hormone (LH), estradiol, testosterone, dehydroepiandrosterone sulphate (DHEAS) and cortisol were also measured. In the men, FSH and partially LH correlated positively with muscle mass percentage, gait speed, handgrip strength and SPPB, and negatively with percent body fat. Additionally, testosterone and DHEAS correlated negatively with the percentage of fat mass in men. Whereas in the women, FSH and LH were mainly negatively associated with body mass and adipose tissue measures. Cortisol did not show any relationship with the examined indicators. The study shows that the indicators of sarcopenia are strongly associated with levels of gonadotropins, sex hormones and DHEAS, especially in older men. The obtained results, after being confirmed in a larger group, may modify prevention and treatment strategies of sarcopenia.

## 1 Introduction

The levels of hormones such as dehydroepiandrosterone (DHEA) and its sulfate (DHEAS), testosterone, cortisol, follicle-stimulating hormone (FSH), luteinizing hormone (LH), and estradiol change with age (1). One aging theory suggests that hormonal changes are related to the progressive aging process (2, 3). Recently, the potential role of gonadotropins (FSH and LH) in age-related diseases has been highlighted (4). It has been found that they may contribute to occurrence of many diseases and syndromes in older life, such as sarcopenia, obesity, frailty syndrome and osteoporosis (5–8). LH may also contribute to mental disability and Alzheimer’s disease (9). FSH, in turn, was shown to increase the risk of cardiovascular diseases (10) and possibly also the risk of cancer development (11, 12). These disorders are key causes of age-related functional deterioration, and thus a loss of independence (13, 14). Numerous publications also indicate a significant relationship between changes in hormone concentrations and occurrence of sarcopenia (15–19). Endocrine diseases, apart from advanced organ dysfunctions, inflammations, and malignant neoplasms, are the main cause of secondary sarcopenia. On the other hand, age-related deficiencies in hormones such as testosterone or DHEAS lead to occurrence of primary sarcopenia (20).

Sarcopenia increases the risk of falls and injuries and deepens the disability and dependence on others (20, 21). It can also be a component of another geriatric syndrome, i.e., frailty syndrome of which effects may be very harmful. The consequences of sarcopenia are frequent institutionalizations and hospitalizations. It generates higher costs of care and imposes a great financial burden on both the state budget and the families of older patients. Sarcopenia also contributes to an increase in mortality (21–23).

A better understanding of the link between hormone levels and muscle mass, physical performance and strength in older subjects may lead to development of possible prevention and treatment methods. Therefore, this study aims to assess the relationship between sarcopenia indicators and circulating gonadotropins and other hormones potentially contributing to age-related functional decline in both male and female seniors.

## 2 Material and Methods

### 2.1 Patients

A group of 100 (39 men and 61 women) outpatients, with a mean age of 80 years, from the Department of Geriatrics of the Medical University of Lodz were recruited. The inclusion criteria were age ≥75 years, being a community-dwelling older adult with functional independence, good verbal and logical communication (without severe dementia), and no signs of malnutrition (MNA>17). The patients who took the hormonal preparations (e.g., thyroxine, corticoids, estrogens, DHEA or testosterone) on recruitment/during the study or who had taken them for the preceding 12 months at least were not included in the study. Similarly, we excluded the patients in whom hypo- and hyperthyroidism, adrenal disorders and other endocrinopathies (except for gonadal failure, which is normal at this age) had been diagnosed.

### 2.2 Methods

All the participants underwent physical examination in the Department of Geriatrics. They were asked about concomitant chronic diseases, including diabetes, cancer, hypertension, heart failure, osteoporosis, history of stroke, and myocardial infarction.

The participants provided basic information on their place of residence. Standard anthropometric measurements (body mass, height) were also performed and Body Mass Index (BMI) was calculated by dividing body mass by height in meters squared.

#### 2.2.1 Body Composition

Body composition, in terms of fat and fat-free mass, was assessed with bioimpedance analysis (BIA) using the AKERN BIA 101 New Edition 50 kHz monofrequency device (AKERN SRL, Florence, Italy). The patients were examined in the horizontal position with electrodes placed on the outside surface of the hand and foot. BIA equipment measures body fat mass however, it does not measure muscle mass directly. Instead, it derives an estimate of muscle mass based on whole-body electrical conductivity. BIA uses a conversion equation that is calibrated with a reference of DXA-measured lean mass. The obtained data were presented as kilograms and the percentage amount of fat mass. The reactance (Xc) and resistance normalized for stature (RI) were used for assessing skeletal muscle mass (SM) and appendicular skeletal muscle mass (ASMM). SM was estimated using the Janssen equation (24). To adjust SM for body size, it was divided by the square of the height (m^2^) and the skeletal muscle mass index (SMI) was obtained. For estimating ASM, the Sergi BIA equation was used, i.e., ASM (kg) = −3.964 + (0.227 × RI) + (0.095 × weight) + (1.384 × sex) + (0.064 × Xc) (25). The ASM Index (ASMI) was defined as ASM/height squared (kg/m^2^) (26).

#### 2.2.2 Levels of Hormones

The blood serum levels of follicle-stimulating hormone (FSH), luteinizing hormone (LH), estradiol, testosterone, dehydroepiandrosterone sulfate (DHEAS), and cortisol were analyzed. Blood samples were taken in the morning from the cubital vein of each patient (overnight fast). Quantitative determinations were performed using technology based on competitive or sandwich chemiluminescence immunoassays (CLIA). The measurements were performed with the use of kits produced by Saluggia (Italy or Stillwater, MN, USA) on a LIAISON XL analyzer from DiaSorin Inc. The expected values and precisions of the assays with the LIAISON XL analyzer are presented in our previous publications (27, 28).

#### 2.2.3 Physical Performance

##### 2.2.3.1 Gait Speed

The participants were asked to walk straight ahead for four meters at their usual pace twice. Walking aids were permitted, however, all the subjects were able to walk independently. The manual measurement was made by trained operators with the use of a stopwatch. The maximal gait speed was used in the analyses and presented in meters per second (m/s) (29).

##### 2.2.3.2 Handgrip

The Jamar hydraulic hand dynamometer (Sammons Preston Rolyan, Bolingbrook, Canada) was used to assess handgrip strength. Each of the participants carried out the handgrip strength test while standing with his/her shoulder adducted and neutrally rotated, and with the elbow in 90 degrees flexion with no radioulnar deviation. There were one-minute rest periods between each attempt, and hands were alternated to minimize fatigue effects. Handgrip strength was measured on the dominant hand twice, with pauses between measurements. The results were recorded as kilogram force. The better result was used for the analysis (30).

##### 2.2.3.3 Chair Stand Test

The participants were asked to cross their arms over the chest and sit with the back against the upright backrest of the chair, then stand up from a chair (seat height was 45 cm) five times, as quickly as possible, without pushing off. The stopwatch was started after the start signal and stopped when the subject sat down on the chair for the fifth time (31).

##### 2.2.3.4 TUG

The Timed Up and Go (TUG) is a simple test used to assess mobility that requires both dynamic and static balance. It measured the time that the patients (wearing their regular footwear and using mobility aids they would normally require) needed to rise from a chair, walk three meters, turn around 180 degrees, walk back to the chair and sit down (32).

##### 2.2.3.5 SPPB

The Short Physical Performance Battery (SPPB) was used to assess physical performance. It consists of three tests to assess the lower extremity function of such items as standing balance in three poses with different difficulty, gait speed at a four‐meter walk, and time to complete five unassisted chair stands. Overall scoring ranges from 0 to 12, with 0 indicating the lowest physical performance, and scores of 12 indicating the highest performance (33, 34).

#### 2.2.4 Statistical Analysis

For a correlation of 0.5 between two variables, the required sample size was found to be 29, and for a correlation of 0.45, the required sample size was found to be 36 (with a power of 0.80 and alpha of.05). The normality of distribution was verified using the Shapiro-Wilk test. Since the variables did not have a normal distribution, they were presented as the median value and quartile range (from the first (25%) to the third (75%) quartile). The quantitative variables between the sexes, were compared using the Mann-Whitney U-test and the qualitative variables using the chi-square test or Fischer’s Exact test. The correlations between the numerical data were analyzed by means of Spearman’s rank correlation coefficient. Multiple linear regression was performed in the men to adjust the potential relationship of performance tests, FSH and LH to body composition and other hormones. The complex relationship of sarcopenia indicators and the hormones (statistically significant in correlations) was assessed, separately in women and men, using multiple stepwise regressions. Variables without normal distributions were log-transformed for these analyses, however, they are presented in standard units. Statistical significance was set at p ≤ 0.05. The analyses were performed using Statistica 13.1 (StatSoft Polska, Cracow, Poland).

#### 2.2.5 Ethical Certification

The study was approved by the Ethics Committee of the Medical University of Lodz (decision no. RNN/363/17/KE of 21^st^ November 2017). Written informed consent was obtained from all the participants.

## 3 Results

**Table 1** presents the general characteristics of the study group according to sex. The median age was 78 for men and 79 years for women. The Mann-Whitney U test showed sex differences between levels of all hormones, all body composition variables, and handgrip strength.

**Table 1:** Characteristics of the study group.

Variables	Men n = 39	Women n = 61	Mann-Whitney U-test/Chi^2^
	Median (quartiles)	Median (quartiles)	P
Age [years]	78 (77-80)	79 (77-81)	ns
Body mass [kg]	77.2 (71.7-84.6)	68 (59-78)	0.0001
BMI [kg/m^2^]	27.9 (25.7-30)	27.4 (24.7-31)	ns
Muscle Mass [kg]	28.2 (26.3-30.4)	17.2 (16.1-19)	<0.0001
Muscle Mass [%]	35.9 (34.9-39.1)	25 (23.8-27.4)	<0.0001
SMI [kg/m^2^]	10.1 (9.6-10.6)	7 (6.6-7.6)	<0.0001
ASM [kg]	22.2 (19.9-23.1)	15.5 (14.2-17)	<0.0001
ASMI [kg/m^2^]	7.73 (7.29-8.16)	6.37 (5.89-6.89)	<0.0001
Fat mass [kg]	18.2 (15.4-22)	24.7 (18.2-29.4)	0.001
Fat Mass [%]	23.9 (20.4-26.1)	35.6 (30.2-38.9)	<0.0001
Gait speed [m/s]	1.15 (1.03-1.28)	1.23 (1.07-1.40)	0.03
Handgrip strength [kg]	36 (31-43)	22 (19.5-25)	<0.0001
Chair Stand Test [sec]	10.5 (9.8-12.5)	11 (10.5-12.6)	0.013
TUG	6.8 (6.4-8.3)	7.35 (6.5-8.4)	ns
SPPB	12 (11-12)	11 (11-12)	ns
FSH [mIU/mL]	12.8 (8.5-23)	87.3 (69-102)	<0.0001
LH [mIU/mL]	6.3 (3.7-10)	19.2 (16.1-25.4)	<0.0001
Estradiol [pmol/L]	113 (95.8-143)	52.6 (45.9-63.2)	<0.0001
Testosterone [nmol/L]	12.1 (9.35-16)	0.75 (0.43-1)	<0.0001
DHEAS [µg/dL]	44.3 (34.9-70.2)	32.6 (22.5-56.3)	0.0033
Cortisol [µg/dL]	16.7 (13.3-21.1)	13.9 (9.55-17.1)	0.02
Diabetes, n (%)	3 (7.69)	11 (18.03)	ns*
Cancer, n (%)	8 (20.5)	9 (14.8)	ns*
Hypertension, n (%)	27 (69.2)	41 (67.2)	ns*
Heart Failure, n (%)	5 (12.8)	18 (29.5)	ns*
Osteoporosis, n (%)	2 (5.1)	23 (37.7)	<0.001*
Stroke, n (%)	4 (10.3)	6 (9.8)	ns*
Myocardial infarction, n (%)	5 (12.8)	2 (3.3)	ns*

ASM, appendicular skeletal muscle mass;

ASMI, appendicular skeletal muscle mass;

SMI, skeletal muscle mass index;

ns, not statistically significant;

DHEAS, dehydroepiandrosterone sulfate;

FSH, follicle-stimulating hormone;

LH, luteinizing hormone;

TUG, Timed up and go test;

SPPB, Short Physical Performance Battery Test;

*Chi^2^ test or Fischer’s Exact test.

Age of the subjects, years of education, BMI, MNA, TUG, and SPPB did not differ significantly. The prevalence of diseases was also similar in both sexes, with the exception of osteoporosis which was more common in women.

The analysis showed that the tested hormones showed few relationships with each other. These correlations were presented in an earlier paper (28). FSH and LH positively correlated with each other in both sex groups. FSH and LH levels were not correlated significantly with estradiol, testosterone, cortisol or DHEAS. Estradiol was positively correlated with testosterone in both sexes, as well as with DHEAS in men.

Two women (5%) had handgrip strength lower than 16 kg while all the men had handgrip strength greater than 27 kg. Eight women (13%) and three men (8%) had the five-repetition chair stand time longer than 15 seconds, two women (3%) and one man (3%) had gait speed slower than 0.8 m/s. A low level of appendicular muscle mass (ASM <15 kg for women; ASM <20 kg for men) occurred in 22 women (36%) and in ten men (26%), however, in the case of four women (7%) and three men (8%) the results were below the definition level of ASMI (for women ASMI <5.5 kg/m^2^; for men ASMI<7 kg/m^2^). None of the subjects needed more than 20 seconds to perform the TUG test, while three women (8%) and none of the men received 8 or fewer points in SPPB.

**Figure 1:**
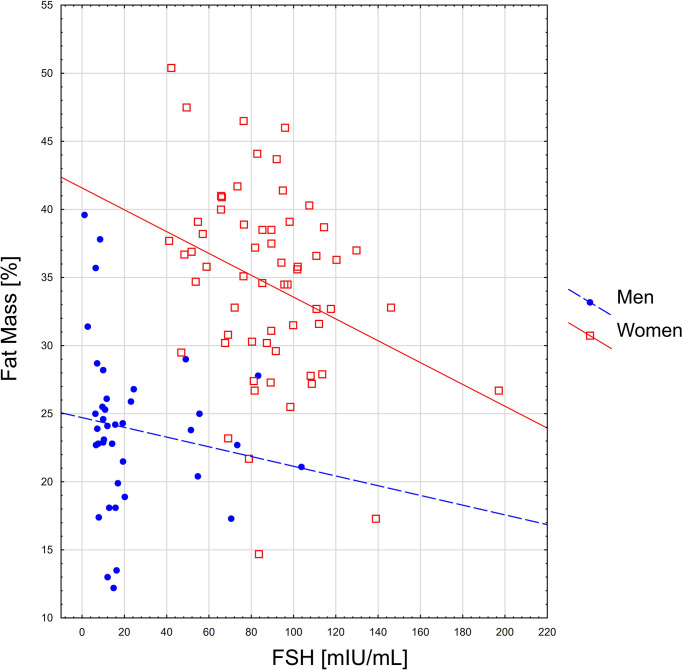
The relationship between FSH level and percentage of fat mass.

**Figure 2:**
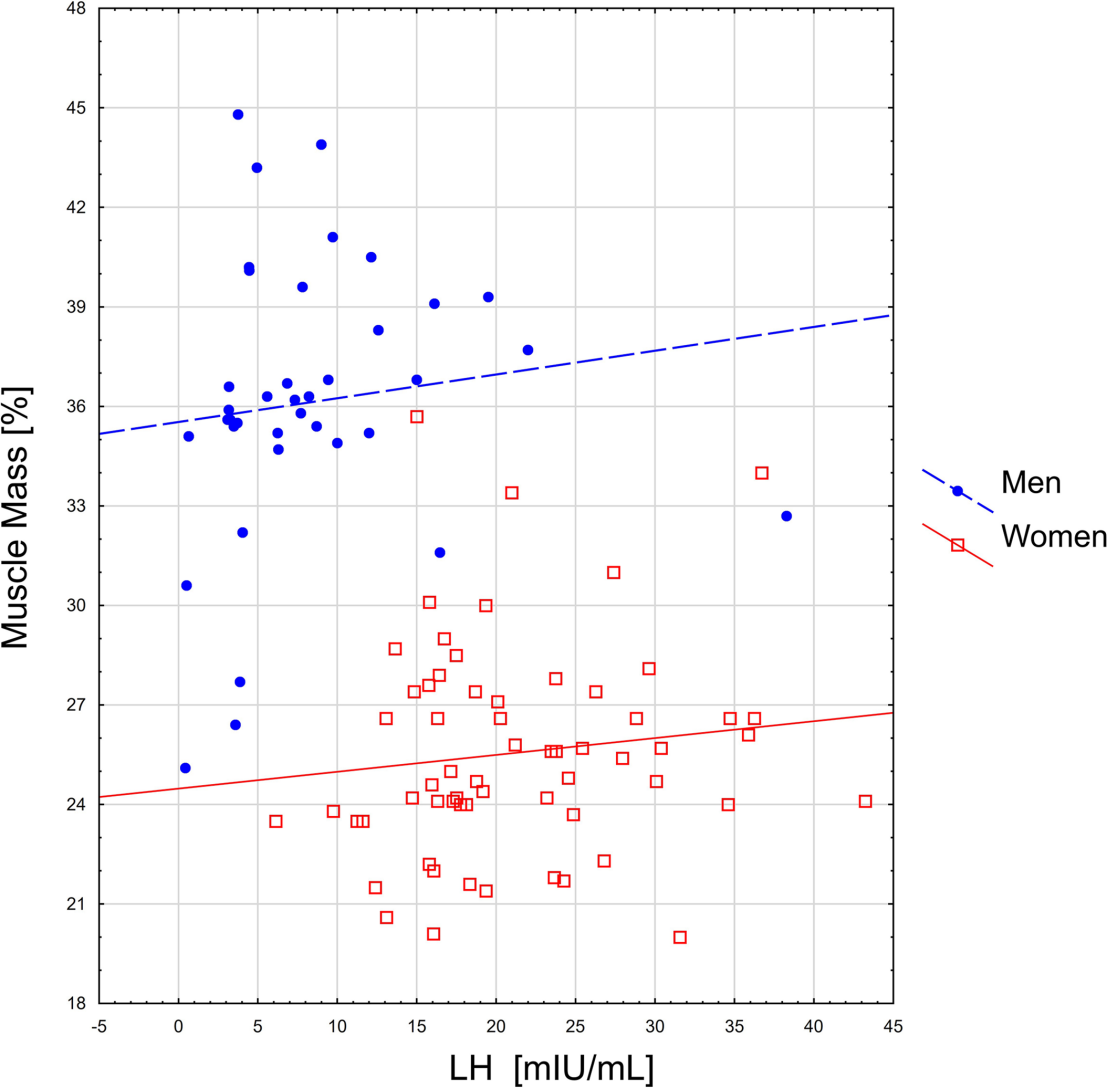
The relationship between LH level and percentage of muscle mass.

Spearman’s rank correlation coefficients between sarcopenia indicators and level of hormones are presented in **Table 2**. The relationship between gonadotropins and selected body components is given in **Figures 1**, **2**.

**Table 2:** Spearman’s rank correlation coefficients of sarcopenia indicators and hormones in men and women groups.

Variables	Spearman’s rank correlation coefficients in men						Spearman’s rank correlation coefficients in women					
	FSH [mIU/mL]	LH [mIU/mL]	Estradiol [pmol/L]	Testosterone [nmol/L]	DHEAS [µg/dL]	Cortisol [µg/dL]	FSH [mIU/mL]	LH [mIU/mL]	Estradiol [pmol/L]	Testosterone [nmol/L]	DHEAS [µg/dL]	Cortisol [µg/dL]
Age [years]	0.213	0.279	0.090	-0.293	-0.015	-0.005	0.023	0.091	0.056	0.053	-0.101	0.335*
Body mass [kg]	-0.268	-0.101	-0.119	-0.270	-0.353*	-0.017	-0.329*	-0.294*	0.167	0.041	-0.069	0.040
BMI [kg/m^2^]	-0.301	-0.144	-0.022	-0.145	-0.314	-0.050	-0.304*	-0.242	0.219	0.068	-0.050	0.025
Muscle Mass [kg]	0.145	0.273	0.249	0.087	-0.231	-0.006	-0.324*	-0.257*	0.041	-0.030	-0.079	-0.100
Muscle Mass [%]	0.387*	0.312*	0.266	0.327*	0.144	0.070	0.163	0.155	-0.125	-0.040	0.132	-0.160
SMI [kg/m^2^]	0.190	0.287	0.366*	0.326*	-0.111	0.001	-0.255*	-0.167	0.184	0.023	0.010	-0.048
ASM [kg]	-0.031	0.110	0.188	-0.068	-0.235	0.082	-0.365*	-0.299*	0.145	0.064	-0.027	-0.040
ASMI [kg/m^2^]	-0.005	0.128	0.303	0.172	-0.168	0.022	-0.330*	-0.226	0.289*	0.115	0.013	-0.009
Fat mass [kg]	-0.322*	-0.160	-0.219	-0.348*	-0.354*	-0.071	-0.307*	-0.271*	0.178	0.048	-0.081	0.064
Fat Mass [%]	-0.340*	-0.191	-0.265	-0.316*	-0.340*	-0.111	-0.267*	-0.256*	0.177	0.038	-0.096	0.080
Gait speed [m/s]	0.336*	0.163	-0.333^*^	-0.039	0.139	0.147	0.062	0.086	-0.034	-0.076	0.058	0.025
Handgrip strength [kg]	0.316*	0.082	0.039	0.007	0.284	0.079	-0.008	0.153	-0.113	0.124	-0.052	-0.007
Chair Stand Test [sec]	-0.323*	-0.108	0.096	-0.085	-0.254	-0.185	-0.039	-0.189	0.126	-0.107	-0.076	-0.157
TUG	-0.004	0.179	0.321*	0.170	-0.014	0.068	0.053	-0.099	0.033	-0.049	-0.095	0.008
SPPB	0.318*	0.155	0.031	0.121	0.231	0.269	0.002	0.016	-0.033	0.082	0.087	0.167

*Correlation statistically significant p<0.05.

ASM, appendicular skeletal muscle mass;

ASMI, appendicular skeletal muscle mass;

DHEAS, dehydroepiandrosterone sulphate;

FSH, follicle-stimulating hormone;

LH, luteinizing hormone;

SMI, skeletal muscle mass index;

SPPB, Short Physical Performance Battery Test

TUG, Timed up and go test

Apart from the direct influence of hormones on the results of performance tests, they may also be affected indirectly through their relationship to body composition or other hormones. Therefore, multiple linear regression was performed in the men to adjust the potential relationship of performance tests, FSH and LH to body composition and other hormones. In multiple linear regression, walking speed remained significantly influenced by FSH and estradiol following adjustment for age, body mass, muscle mass or testosterone level. FSH was not a statistically significant predictor of handgrip strength or chair stand test following adjustment for age or muscle mass. Estradiol was not an independent predictor of TUG. In the men, SPBB remained significantly influenced by FSH after adjustment for age or muscle mass, however, not following adjustment for body mass, muscle mass % or testosterone level.

**Figure 3:**
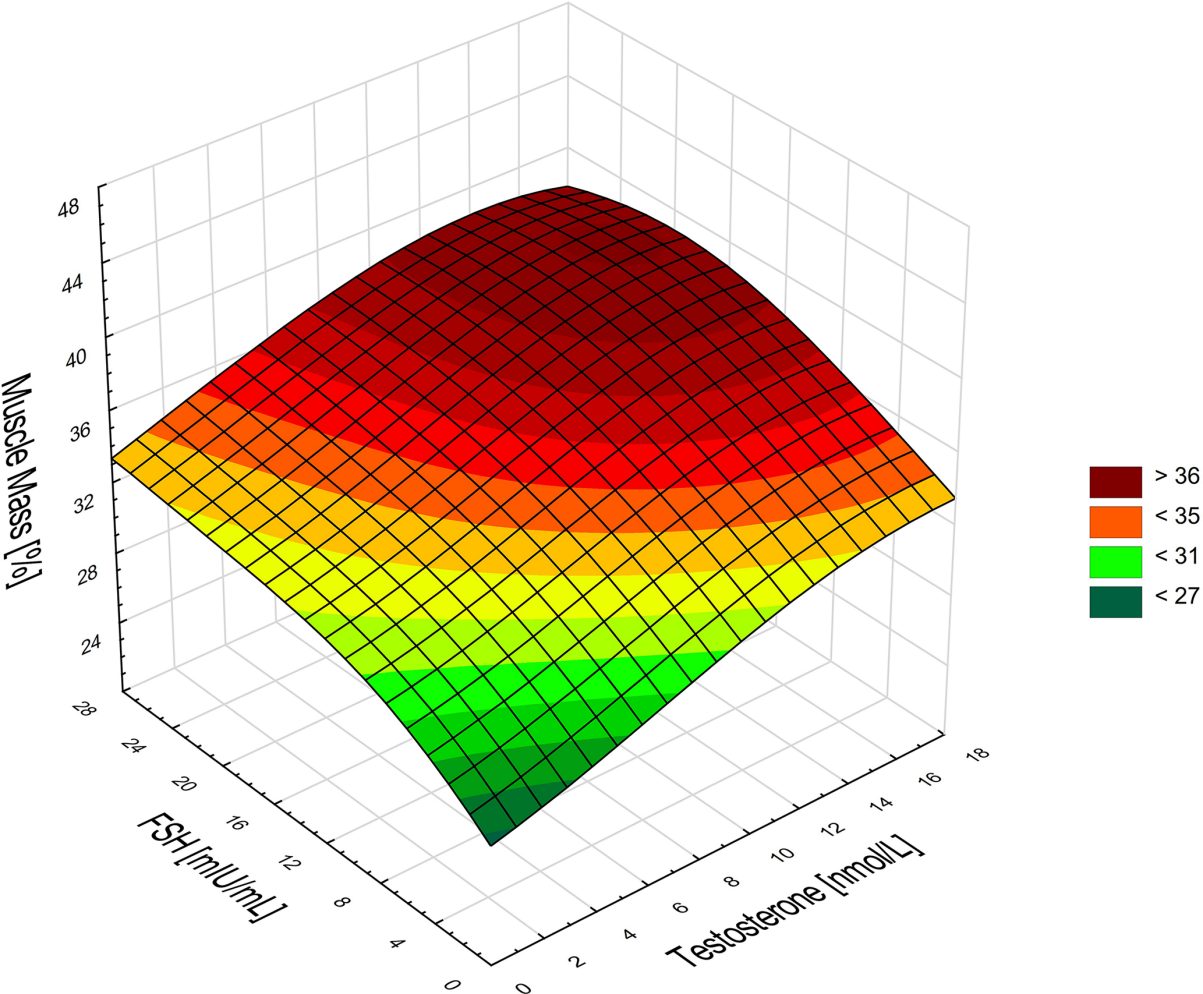
Associations between the percentage of muscle mass, FSH, and testosterone in men. Higher FSH and testosterone levels are associated with a higher percentage of muscle mass in men.

The results of the performed stepwise regression confirm that in men the level of FSH (β=0.29; p=0.044) and testosterone (β=0.42; p=0.005) were the best determinants of the percentage of muscle mass as a dependent variable (R^2^ = 0.31). In order to improve visualization of these cumulative effects of the variables, they are presented as a surface chart (**Figure 3**).

In the men, SMI was described by estradiol (β=0.468; p=0.03; R^2^ = 0.22), whereas in the women by FSH (β=-0.335; p=0.008; R^2^ = 0.293).

Similarly, in the women, the interaction of FSH (β=-0.33; p=0.008) and estradiol (β=0.24; p=0.048) was the strongest determinant of ASMI as the dependent variable (R^2^ = 0.19). For ASM, the determinants were FSH (β=-0.46; p<0.0001) and age (β=-0.31; p=0.007), R^2^ = 0.314.

In the women, the percentage of body fat (β=-0.33; p=0.01; R^2^ = 0.11) was best described by FSH. While in the men, it was the interaction of testosterone (β=-0.4; p=0.008), and DHEAS (β=-0.31; p=0.04) that determined the percentage of body fat (R^2^ = 0.28) best.

In the men, FSH (β=0.38; p=0.015) and estradiol (β=-0.34; p=0.027) best described the gait speed (R^2^ = 0.18). There was no statistically significant association for the rest of the physical performance tests and hormones in multiple stepwise regression.

## 4 Discussion

To the best of our knowledge, this is the first paper to provide a detailed analysis of the relationship between sarcopenia indicators and the level of gonadotropins, testosterone, estradiol, DHEAS, and cortisol in older adults. Several important associations have been identified and, interestingly, some sex-related differences were observed in those relationships. The study population comprised properly-nourished senior citizens, without a disability, presenting hormonal changes typical for their age. The mutual relationships between the tested hormones have been described in our previous publications (27, 28).

The indicators of sarcopenia have been chosen according to the definition and diagnostic criteria for the disease, as proposed by the second European Working Group on Sarcopenia in Older People (EWGSOP2) (20). Based on them, sarcopenia can be diagnosed in four women and two men, whereas, low muscle mass in one-third of the women and one-quarter of the men.

The relationship between the muscle indexes SMI and ASMI is different from that occurring in the case of muscle mass percentage or fat mass. This is especially true for women. Additional correlation analyses showed that both ASMI and SMI were strongly correlated with body fat content. In men, SMI positively correlates with muscle mass percentage (rS=0.39; p<0.05), and in women there is no such a correlation, however, there is a positive association with BMI (rS=0.59, p<0.05). On the other hand, ASMI in men is not correlated with muscle mass percentage but with BMI (rS=0.69; p <0.05). In women, the strongest positive correlations are with BMI (rS=0.89; p<0.05) and adipose tissue percentage (rS=0.61; p<0.05). Surprisingly, the relationship of ASMI with muscle mass percentage is opposite (rS=-0.35; p<0.05). Similar conclusions were presented by Shimizu et al., who suggest that in older people with a high body fat content, indices of muscle mass divided by the square of the height are not appropriate (35).

Among the observed correlations, the most important one seems to be the relationship between higher FSH and a higher percentage of muscle mass in men. In both sexes, higher level of gonadotropins were related to lower body fat percentage. Additionally, it has been observed that in men this phenomenon is accompanied by higher testosterone and DHEAS levels.

In the presented analysis, higher concentration of gonadotropins seems to have a protective effect on muscle mass. However, in other studies, unfavorable effects of FSH and LH have been described. Pawlikowski et al. suggest that a high level of these hormones is an essential element of aging (especially in postmenopausal women). It should be assumed that the direct effects of gonadotropins, whose receptors are found in different other organs and tissues outside the reproductive system, may be involved in the pathogenesis of several age-related disorders (4, 36). However, those papers did not analyze the relation of gonadotropins with the musculoskeletal system.

In mice, blocking the action of FSH increases bone mass and decreases fat mass (36). A recent rodent study by Liu et al. raised a possibility that increased levels of circulating FSH could be another strong factor mediating the decline in skeletal muscle mass during the menopausal transition (36). Park et al. suggest that in a late perimenopausal period associated with an increase in FSH levels, women may be prone to skeletal muscle loss (6). In a cross-sectional study including 141 healthy women classified as premenopausal (38 years, n=30), early perimenopausal (50 years, n = 31), late perimenopausal (50 years, n=30), early postmenopausal (55 years, n=24), or late postmenopausal (62 years, n=26), the menopause-related loss of spine and hip bone mineral density was associated not only with low estrogen but also higher FSH (7). In the cross-sectional AGES-Reykjavik Study conducted among women (n = 238, mean age 81 years), those in the highest FSH quartile, as compared to those in the lowest one, had a lower spine integral bone mineral density, lower weight, lower visceral adipose tissue, lower lean mass, and lower fat mass. In men, FSH level was not associated with any outcome (5).

Although a lower estradiol level is believed to be the most important factor in the menopause-related decline in muscle mass, the quoted authors suggest that it is also related to elevated FSH levels. The results reported in the present study seem to be contradictory to the data obtained by Park and colleagues. However, the women surveyed by the said authors were much younger than those examined in our study. A similar discrepancy of FSH levels in relation to age (early postmenopausal vs. very advanced age) seems to occur in the case of a relation of FSH to the fat tissue mass, reported both by Liu et al. and in our study (28, 37). Likewise, in a three-year prospective study of 162 women and 158 men (mean age 82 years), there was no evidence for an association between the baseline FSH level and change in bone mineral density (BMD) and body fat (38). To explain these discrepancies, we have suggested that the function of FSH receptors may be shifted from lipogenesis to lipolysis at a more advanced age (28). A similar (although not identical) mechanism could be considered in the case of FSH influence on skeletal muscles. However, this presumption needs further studies to be proved. Liu et al. reported on the presence of FSH receptors in skeletal muscles (36). This observation allows to presume the direct action on the muscles, however, the precise mechanism of this action remains unknown.

There is a strong clinically important relationship between decreased levels of androgens and estrogens occurring at older age, age-related decline in muscle and bone mass, and strength (39). Numerous studies describe the relationship between testosterone deficiency and decrease in muscle mass and strength, bone mass, and an increase in visceral adipose tissue in older men (40, 41). Serum testosterone concentration below the level considered as normal for young men occurs in approximately 30% of men aged over 70 years and half of men aged over 80 years (42). A lower concentration of sex hormones is not only a change in body composition but also a greater risk of frailty syndrome (43). In the women participating in our study, the percentage of fat mass was not related to either testosterone or estradiol, while in the men there was a negative trend. Additionally, in the men, a higher testosterone level was associated with a higher percentage of muscle mass and a higher SMI. These results confirm that obesity and sarcopenia in men may be associated with low testosterone level.

The relationship between the concentration of hormones and results of physical performance tests did not show such a strong correlation as in the case of the body composition measurements. However, their direction generally coincided with the expected relationships. The tests we used, i.e. walking speed, handgrip strength, getting up from a chair, TUG and SPPB, are commonly applied indicators of physical functioning used in geriatric medicine.

The strongest associations were observed with gonadotropins, especially with FSH in the men, whereas in the women there was no such relationship. So far, scientific publications have not assessed this type of a relationship. Nevertheless, some researchers point to the relationship of physical performance with DHEAS and testosterone. When the secretion of DHEAS is maintained in the elderly, it entails a lower risk of ailments of frailty and physical weakness, assessed as handgrip strength, repetitive chair stands and gait speed (44). In the Cardiovascular Health Study, DHEAS was independently associated with the handgrip strength (45). In the oldest old women, such correlation wasn’t observed, but a decrease in DHEAS was associated with a decrease in walking speed (46). On the other hand, a lower circulating DHEAS level was related to lower leg extensor power in the older women, not in the men, though (47).

An analysis of the population of older men from the InCHIANTI study showed that testosterone levels ≤230 ng/dL were (regardless of age and BMI) associated with lower handgrip strength and poorer SPPB results. However, there was no relationship between testosterone and calf muscle mass and walking speed (48). Schaap et al. did not observe any deterioration of performance, assessed as the handgrip strength, with a decrease in testosterone levels during the three-year follow-up either (49). In our study, the relationships with testosterone and DHEAS were not statistically significant, although their direction was consistent with that found by other researchers. This may be due to a relatively small group of participants. In our study, no relationship between cortisol and physical performance was identified, while some other studies showed a negative relationship with high cortisol levels in the elderly (50). The meta-analysis also proved that greater diurnal decline of the HPA axis is associated with better physical performance in the elderly (51).

### 4.1 Limitations

It should be acknowledged that the study was conducted in a group of volunteers from the Central Europe and all of them were Caucasian. The study is a pilot study and was designed for a relatively small group of people (100 participants). The obtained results should be confirmed in a larger, more representative population. People with advanced dementia, seriously ill, or disabled were not included in the study. Therefore the relationships observed in the study group may be different in other populations. Finally, in such studies there is always a question of correction for multiple comparisons. We did not apply Bonferroni corrections to avoid Type II error or misinterpretation of the potential relationship of multiple sarcopenia indicators with a given hormone.

## 5 Conclusions

From among the relationships found in the study, the following three were the most important ones:

1) A positive correlation between gonadotropins and the percentage of muscle mass;

2) An association between the lower percentage of fat mass and higher levels of gonadotropins in both sex groups and higher testosterone and DHEAS in men;

3) A positive correlation between FSH and some physical performance tests in men.

The study shows that the indicators of sarcopenia are strongly associated with levels of gonadotropins, sex hormones, and DHEAS, especially in older men. Cortisol did not show any relationship with the examined indicators. The obtained results, after being confirmed in a larger group, may become an element of prevention and treatment of sarcopenia.

## Data Availability Statement

The raw data supporting the conclusions of this article will be made available by the authors, without undue reservation.

## Ethics Statement

The studies involving human participants were reviewed and approved by Ethics Committee of the Medical University of Lodz, decision no. RNN/363/17/KE of 21st November 2017. The patients/participants provided their written informed consent to participate in this study.

## Author Contributions

Conceptualization, AG. Methodology, AG, MPa, KW, and TK. Validation, AG. Formal Analysis, AG. Investigation, AG, ZC, MPa, MPi, HP, KW, and TK. Resources, AG, ZC, MPi, HP, and TK. Data Curation, AG. Writing – Original Draft Preparation, AG and ZC. Visualization, AG. Supervision, TK. Funding Acquisition, TK. All authors contributed to the article and approved the submitted version.

## Funding

This work was supported by the Medical University of Lodz [grant number 503/6-077-01/503 61-001].

## Conflict of Interest

The authors declare that the research was conducted in the absence of any commercial or financial relationships that could be construed as a potential conflict of interest.

## Publisher’s Note

All claims expressed in this article are solely those of the authors and do not necessarily represent those of their affiliated organizations, or those of the publisher, the editors and the reviewers. Any product that may be evaluated in this article, or claim that may be made by its manufacturer, is not guaranteed or endorsed by the publisher.
